# Comparison of Postoperative Hyphemas between Microhook Ab Interno Trabeculotomy and iStent Using a New Hyphema Scoring System

**DOI:** 10.3390/jcm10235541

**Published:** 2021-11-26

**Authors:** Akiko Ishida, Sho Ichioka, Yuji Takayanagi, Aika Tsutsui, Kaoru Manabe, Masaki Tanito

**Affiliations:** Department of Ophthalmology, Faculty of Medicine, Shimane University, Izumo 693-8501, Japan; ishidaki@med.shimane-u.ac.jp (A.I.); sho-ichi.1002@med.shimane-u.ac.jp (S.I.); y.takayanagi1008@med.shimane-u.ac.jp (Y.T.); aika0408@med.shimane-u.ac.jp (A.T.); manabe42@med.shimane-u.ac.jp (K.M.)

**Keywords:** minimally invasive glaucoma surgery (MIGS), Tanito microhook (TMH), iStent trabecular micro-bypass system, surgical efficacy, surgical complication, hyphema

## Abstract

We have been using our in-house scoring system of hyphemas, i.e., Shimane University RLC postoperative hyphema scoring system (SU-RLC), which we designed to classify postoperative hyphema. SU-RLC classifies the severities of hyphemas based on three factors, i.e., red blood cells (RBCs) (R) 0–3, layer formation (L) 0–3, and clot (C) 0–1, by slit-lamp observation. To test the clinical usefulness of the SU-RLC for quantifying the postoperative hyphema severity, the SU-RLC scores were compared between eyes that underwent different minimally invasive glaucoma surgery (MIGS) procedures, i.e., Tanito microhook ab interno trabeculotomy and cataract extraction (TMH-CE) (*n* = 64 eyes of 64 subjects; mean age ± standard deviation, 72.4 ± 8.1 years) and iStent-CE (*n* = 21 eyes of 21 subjects; 76.1 ± 10.6 years). Compared to the iStent-CE, higher hyphema scores with the TMH-CE were found for the R scores on postoperative days 1, 2, and 3; for the L score on postoperative day 1; and for the C score on postoperative day 2. The sums of the R, L, and C scores (RLC) on postoperative day 1 were 3.2 ± 1.1 with the TMH-CE and 1.1 ± 1.3 with the iStent-CE; the scores reached almost 0 by 2 weeks in both groups. Significant differences in the RLC scores between the surgical groups were found on postoperative days 1, 2, and 3. Multivariate analyses showed that the TMH-CE rather than iStent-CE was associated with higher R, C, and RLC scores; anticoagulant/antiplatelet use was associated with higher R score; and myopia was associated with a higher C score. In the TMH-CE group, myopia was associated with a higher C score. In the iStent-CE group, anticoagulant/antiplatelet use was associated with higher R and RLC scores; and higher postoperative 1-day intraocular pressure was associated with a higher C score. The SU-RLC successfully detected the difference in hyphema severity between different MIGS procedures; thus, we concluded that our classification system may be feasible to evaluate hyphemas after glaucoma surgery.

## 1. Introduction

Postoperative hyphema is a common complication during glaucoma surgeries, including trabeculectomy, tube-shunt surgeries, and minimally invasive glaucoma surgeries (MIGS) [1,2]. Traditionally, the severity of postoperative hyphemas has been described using the classification for traumatic hyphemas [3]. This classifies hyphemas into three grades including layer formation less than 1/3 of the anterior chamber (AC) as grade I, 1/3 to 1/2 of the AC as grade II, and greater than 1/2 of the AC including an eight-ball hemorrhage as grade III [3]. This can be useful to classify severe hyphemas; however, given that most hyphemas seen after glaucoma or ocular surgeries are milder than grade I, it may be unsuitable to quantify surgically induced hyphemas. Other researchers have quantified postsurgical hyphemas by using a classification system based on the AC cells seen in eyes with uveitis [4,5]. This classification quantifies the AC cells by the number of cells seen in the field of a 1-mm by 1-mm slit beam as <1, 1–5, 6–15, 16–25, 26–50, and >50 cells/field for respective grades of 0, 0.5+, 1+, 2+, 3+, and 4+ [6]. This is useful to quantify the number of inflammatory cells; however, given that the number of red blood cells (RBCs) in blood is >500 times greater than the white blood cells, counting the RBCs using this classification is technically difficult in eyes postoperatively. Other than the presence of the layer formation and floating RBCs in the AC, blood clots frequently seen postoperatively cannot be described using those previously described classifications.

Since 2019, we have been using our in-house scoring system, which we refer to as the Shimane University RLC postoperative hyphema scoring system (SU-RLC). We designed the SU-RLC specifically to classify postsurgical hyphemas; in this system, the levels of floating RBCs, the height of the formed layer, and the presence/absence of blood clots were classified separately, as will be described.

Postoperative hyphema is one of the most frequent complications occurring after goniotomy procedures [7], but development of hyphemas is relatively rare after use of the iStent trabecular micro-bypass system [8]. In the current study, to test the clinical usefulness of the SU-RLC to quantify the postsurgical hyphema severity, the SU-RLC scores were compared between eyes treated with two MIGS procedures: Tanito microhook ab interno trabeculotomy and cataract extraction (TMH-CE) and iStent-CE. Postsurgical hyphema can be affected by use of systemic anticoagulants and antiplatelets [9] and can be associated with postoperative intraocular pressure (IOP) increases [4]. Therefore, we also assessed the association between the hyphema scores and various parameters.

## 2. Subjects and Methods

### 2.1. Subjects and Data Collection

The study adhered to the tenets of the Declaration of Helsinki; the institutional review board (IRB) of Shimane University Hospital reviewed and approved the research (No. 20200501-1). The IRB approval did not require that each patient provide written informed consent for publication; instead, the study protocol was posted at the study institution to notify participants about the study. This retrospective observational case series included consecutive 85 eyes of 85 Japanese subjects (39 men, 46 women; mean age ± standard deviation (SD), 73.3 ± 8.8 years). All subjects who fulfilled the inclusion criteria and did not meet the exclusion criteria were selected from the department database. The inclusion criteria included eyes that underwent TMH-CE or iStent-CE performed by one surgeon (MT) between September 2019 and August 2020; the eyes had primary open-angle glaucoma (POAG) or exfoliation glaucoma (EXG); no history of previous intraocular surgery; and completed full postoperative visits on postoperative days 1, 2, 3, 2 weeks (range, 1–2 weeks), and 3 months (2–4 months). In addition, the hyphema severity scored based on the SU-RLC was recorded in the medical chart or anterior-segment photographs that enabled determination of the SU-RLC scores were taken at every follow-up visit. The exclusion criteria included an additional simultaneous procedure during the TMH-CE or iStent-CE; any intraoperative complication including posterior capsular rupture, Zinn’s zonular dialysis, or goniodialysis; and any intervention within 3 months after TMH-CE or iStent CE were performed. If both eyes were eligible, the eye with the earlier surgical day was included. The following data were collected during a chart review: Age, gender, glaucoma type, preoperative use of an anticoagulant or antiplatelet therapy, preoperative spherical equivalent refractive error (SERE), preoperative and postoperative IOP values and number of antiglaucoma medications, and postoperative SU-RLC scores. The IOP was measured using the iCARE rebound tonometer (M.E. Technica, Tokyo, Japan) on postoperative days 1, 2, and 3 and by Goldmann applanation tonometer at postoperative 2 weeks and 3 months.

### 2.2. Surgical Procedures

Before the TMH or iStent implantation, phacoemulsification cataract surgery was performed through a 2.2-mm-wide clear corneal incision created at the 9 to 10 o’clock position (i.e., temporal incision for the right eye and nasal incision for the left eye); a one-piece soft acrylic intraocular lens was inserted into the capsular bag through the same clear corneal incision. In cases that underwent a TMH procedure, spatula-shaped microhooks (M-2215S, 2215R, and 2215L, Inami, Tokyo, Japan) designed specifically for use during the TMH procedure then were used [7,10]. Viscoelastic material (1% sodium hyaluronate, Provisc, Alcon Japan, Tokyo, Japan) was injected into the AC through the clear corneal ports created using a 20-gauge micro-vitreoretinal knife (Mani, Utsunomiya, Japan) at the 2 to 3 and 9 to 10 o’clock positions. A microhook was inserted into the AC through the corneal port, and a Swan-Jacob gonioprism lens (Ocular Instruments, Bellevue, WA, USA) was used to observe the angle opposite to the corneal port. The microhook tip then was inserted into Schlemm’s canal and moved circumferentially to incise the inner wall of Schlemm’s canal and trabecular meshwork (TM) over 3 clock hours. Using the same procedure, LOT was performed in the opposite angle using a microhook inserted through the other corneal port. Accordingly, the LOT extended more than half of the circumference. In cases that underwent iStent implantation under observation using a Swan-Jacob gonioprism lens, a first-generation iStent device (GTS100R for right eyes and GTS100L for left eyes, Glaukos Japan, Tokyo, Japan) was implanted into Schlemm’s canal through the TM at the inferonasal quadrant. After TMH or iStent implantation, the viscoelastic material was aspirated, and the corneal ports were closed by corneal stromal hydration. At the end of surgery, a steroid (2 mg of betamethasone sodium phosphate, Rinderone, Shionogi Pharmaceutical, Osaka, Japan) was injected subconjunctivally and 0.3% ofloxacin ointment (Tarivid, Santen Pharmaceutical, Osaka, Japan) was applied. Finally, 1.5% levofloxacin (Nipro, Osaka, Japan) and 0.1% betamethasone (Sanbetason, Santen Pharmaceutical) were applied topically four times daily for 3 to 4 weeks postoperatively in all cases. An anticoagulant or antiplatelet used before surgery was not discontinued perioperatively.

### 2.3. SU-RLC Postoperative Hyphema Scoring System

**Figure 1:**
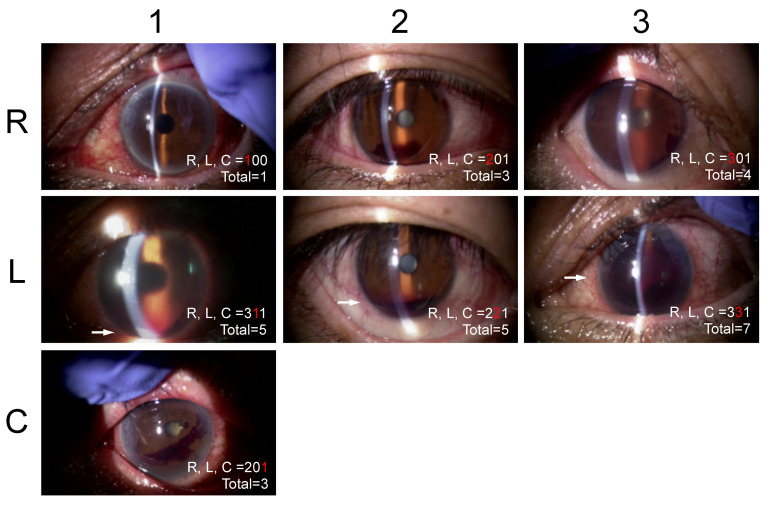
Representative anterior-segment photographs of each R, L, and C score. The Shimane University RLC postoperative hyphema scoring system can be expressed as a three-digit number when the clinicians record the hyphema severity in the medical chart. (**R**) Red blood cells score; (**L**) layer formation score; (**C**) blood clot score.

This system classifies the severities of hyphemas based on three factors including the red blood cells (RBCs) (R), layer formation (L), and clots (C) by slit-lamp observation (Table 1, Figure 1). R is categorized as 0 indicating no floating RBCs in the AC, 1 floating RBCs are seen but iris patterns are observed clearly in the entire AC, 2 floating RBCs are seen but unclear iris patterns are observed, and 3 dense floating RBCs are seen and iris patterns are not observed. L is categorized as 0 indicating no L, and 1 less than 1 mm high or 2 central corneal thickness, and 2 below the inferior pupillary margin, and 3 above the inferior pupillary margin. C is categorized as 0 indicating no blood clots in the AC and 1 the presence of blood clots. The scores recorded in the medical charts were collected. When the score was not recorded, one author (AI) determined a score based on the stored anterior segment photographs. The agreement in the RLC scores between two scorers (MT and AI) in 20 random anterior-segment photographs was calculated to be excellent for R (kappa = 0.8387, by Cohen’s kappa statistics), L (0.9103), and C (0.8750) and substantial for the RLC (0.7538) based on the agreement classification proposed by Altman et al. [11].

### 2.4. Statistical Analysis

The data were compared between the TMH-CE and iStent-CE groups using the un-paired *t*-test for continuous variables and Fisher’s exact probability test for categorical variables. To assess possible associations between the RLC score and various background parameters, correlations between the RLC score and continuous variables were tested using Pearson’s correlation coefficient test, and the RLC scores were compared between categorial groups using the unpaired *t*-test. The parameters associated with the RLC score was assessed further by multiple regression analysis. All continuous data are expressed as the mean ± standard deviation (SD). All statistical analyses were performed using the JMP Pro version 14.2 statistical software (SAS Institute, Inc., Cary, NC, USA). *p* < 0.05 was considered significant. By power calculation, when the alpha error was set at 0.05, the statistical power for detecting differences in the mean RLC score of 1.0 with a SD of 0.5 was calculated to be >0.99 with the sample sizes of the current data set (i.e., 64 in TMH-CE and 21 in iStent-CE groups).

## 3. Results

The demographic data of the subjects are summarized in Table 2. The preoperative IOP and medications were significantly higher in the TMH-CE group than in the iStent-CE group, while other parameters including age, gender, glaucoma type, SERE, and anticoagulant/antiplatelet use were equivalent between the surgical groups.

The IOP, medication, and RLC scores during the follow-up period are summarized in Table 3. The postoperative IOP was significantly higher in the TMH-CE group than in the iStent-CE group at postoperative days 1 and 2, and the medication number was higher in the TMH-CE group than in the iStent-CE group at all follow-up periods for up to three months. Compared to iStent-CE, higher hyphema scores in the TMH-CE group were found for the R score on postoperative days 1, 2, and 3; for the L score on postoperative day 1; and for the C score on postoperative day 2. The sum of the R, L, and C scores on postoperative day 1 was 3.2 in the TMH-CE group and 1.1 in the iStent-CE group; the scores became almost 0 by two weeks in both groups. Significant differences were seen between the surgical groups in the RLC scores on postoperative days 1, 2, and 3. 

Univariate analyses performed to identify possible associations between the RLC sores recorded on postoperative day 1 and various continuous parameters are summarized in Table 4. Significant associations were found between higher R scores and higher preoperative IOPs and more medications; between higher L scores and more preoperative medications and higher postoperative one-day IOP while larger postoperative three-month IOP reduction; between higher C scores and younger age, myopic SERE, and higher postoperative three-month IOP. Younger age, higher preoperative IOP and more medications, and higher postoperative one-day IOP were associated significantly with higher RLC scores.

Univariate analyses to identify possible associations between the RLC sores recorded on postoperative day 1 and various categorical parameters are summarized in Table 5. Male gender was associated with a higher L score, while the scores did not differ significantly between POAG and EXG or between use and no use of anticoagulant/antiplatelet therapies.

Multivariate analyses performed to identify possible associations between the RLC sores recorded on postoperative day 1 and various parameters are summarized in Table 6. TMH-CE and not iStent-CE was associated with higher R, C and RLC scores; and myopic SERE was associated with higher C scores.

Multivariate analysis results in each surgical group are summarized in Table 7 and Table 8. In the TMH-CE group, younger age and myopic SERE were associated with higher C scores (Table 7). In the iStent-CE group, anticoagulant/antiplatelet use was associated with higher R and RLC scores; and higher postoperative one-day IOP was associated with higher C score (Table 8).

## 4. Discussion

We found that the R, L, C, and/or RLC scores were higher in the TMH-CE group than in the iStent-CE group for up to three days postoperatively, while the scores became almost 0 by two weeks in both surgical groups. Multivariate analyses showed that differences in surgical procedures, anticoagulant/antiplatelet use, and myopic SERE were associated with higher R, C, or RLC scores. The classification system that separately estimated each component of hyphema (i.e., R, L, and C) is unique in the literature. The agreement in the RLC scoring between two scorers was between excellent and substantial (Section 2.3). In daily practice, we can express the severity of each component of hyphemas as a three-digit number, e.g., 221 for R = 2, presence of floating RBCs; iris patterns observable but unclear; L = 2, layer formation below inferior pupillary margin; and C = 1, presence of blood clots. We believe that use of the SU-RLC helps clinicians quantitatively describe the hyphemas during the postoperative periods.

In the current study, the IOP was higher in the TMH-CE group than in the iStent-CE group on postoperative days 1 and 2; this may be explained by the presence of more hyphemas in the former than the latter since the postoperative one-day IOP was significantly associated with the RLC score (Table 4). The association between floating RBCs in the AC and IOP also was evidenced by the delayed hyphemas seen after use of the Trabectome (NeoMedix Corp., Tustin, CA, USA) [4] and TMH [12]. In this study, the IOPs were the same between two surgical groups on postoperative day 3 and later for up to three months; this also coincided well with the reduction in the RLC score in this dataset. In a previous fellow-eye comparison, we reported that there was no significant difference in IOP between postoperative two weeks and 12 months postoperatively, while reductions in the IOP were greater in the TMH-CE group than in the iStent-CE group at three months postoperatively and later [13]. The postoperative one-day RLC score was not associated with the postoperative IOP and medications at three months (Table 4). Thus, our results suggested that higher RLC scores during the early postoperative period were not the predictors of the surgical efficacy after MIGS surgeries. Among the R, L, and C scores, the L score was associated with larger IOP reduction at three months. This score might reflect the integrity of post-Schlemm’s canal outflow [14], but this requires to be tested. The C score was associated with higher postoperative IOP at 3 months. This score might affect later IOP levels via peripheral anterior synechia formation [15], although this remains to be determined in the future. 

In the TMH-CE group, myopic SERE was associated with a higher C score (Table 7). By univariate analysis, younger age was associated with a higher C score (Table 4). We previously reported cases of postoperative hypotony after use of the TMH; myopia and young age were the common features in those cases [16]. The pectinate ligament, also referred to as the ciliary process or mesodermic remnant, can regress during aging [17]. The TM in glaucomatous eyes is characterized by decreased elasticity and increased stiffness as a result of aging [18,19]. Myopia is the common feature of juvenile-onset glaucoma [20]. Thus, traction on the pectinate ligament or elastic TM tissue exerted by a relatively dull device (i.e., microhooks) may explain the bleeding from the angle and blood clot formation in young patients with glaucoma.

Anticoagulant/antiplatelet use was associated significantly with higher R scores (Table 6); the association was significant for the R and RLC scores in the iStent-CE group (Table 8) and not so in the TMH-CE group (Table 7). Anticoagulant/antiplatelet use was associated with a significant increase in the rate of hemorrhagic complications after tube shunt, trabeculectomy, and trabeculectomy-CE [9]. Hyphema is by far the most common complication after goniotomy procedures compared with other glaucoma surgeries [2,8]; thus, the effects of anticoagulant/antiplatelet use on hyphemas might have been obscured in the TMH-CE group. Given that anticoagulant/antiplatelet use seemed to not be associated with prolonged hyphemas, the current results do not support the requirement of presurgical cessation of these medications before iStent procedures.

While we included all the subjects who fulfilled the inclusion and exclusion criteria, the retrospective nature of this study may have caused selection bias. While the study sample size was not predetermined, the statistical power calculated (>0.99) was sufficiently strong to detect differences in the RLC scores between the surgical groups. In the statistical analysis, we used the sum of the R, L, and C scores as the general indicator of hyphema severity. Since these numbers were not coordinated each other, different calculation methods to express the total hyphema severity may exist, but this needs to be investigated further. Since the primary purpose of this study was to test the usefulness of the SU-RLC in eyes after glaucoma surgery, we excluded eyes with intraoperative complications and any intervention including AC washout within three months postoperatively. Severe hyphemas can be associated with transient IOP spikes; thus the associations between the postoperative IOP and hyphema severity might have been underestimated in this study. Future prospective and multicenter studies by including various types of glaucoma surgeries can be helpful to generalize this scoring system.

## 5. Conclusions

The SU-RLC system successfully detected the difference in hyphema severity between different MIGS procedures. We concluded that our classification system may be feasible for evaluating hyphemas after glaucoma surgery.

## Figures and Tables

**Table 1:** Shimane University RLC postoperative hyphema scoring system.

Score	0	1	2	3
Red Blood Cells (R)	No floating RBCs in AC	Floating RBCs; iris patterns clearly seen in entire AC	Floating RBCs; iris patterns seen but not clearly	Dense floating RBCs; no iris patterns seen
Layer formation (L)	No layer formation	Layer formation lower than 1 mm (2 CCT) height	Layer formation below inferior pupillary margin	Layer formation above inferior pupillary margin
Clots (C)	No blood clots	Blood clots present		

RBCs—red blood cells; AC—anterior chamber; CCT—central corneal thickness.

**Table 2:** Demographic data.

Parameter	Total	TMH-CE	iStent-CE	*p*-Value
N, eyes	85	64	21	
Age, years				
Mean ± SD	73.3 ± 8.8	72.4 ± 8.1	76.1 ± 10.6	0.0952
95% CI	71.4, 75.2	70.4, 74.4	71.3, 80.9	
Gender				
Male, *n* (%)	39 (46)	30 (47)	9 (43)	0.8048
Female, *n* (%)	46 (54)	34 (53)	12 (57)	
Glaucoma type				
POAG, *n* (%)	59 (69)	41 (64)	18 (86)	0.0998
EXG, *n* (%)	26 (31)	23 (36)	3 (14)	
Preoperative IOP, mmHg				
Mean ± SD	18.7 ± 5.1	19.7 ± 5.3	15.9 ± 3.3	0.0030 **
95% CI	17.6, 19.8	18.3, 21	14.4, 17.4	
Preoperative medications				
Mean ± SD	2.5 ± 1.1	2.8 ± 0.9	1.6 ± 1.0	<0.0001 **
95% CI	2.3, 2.8	2.6, 3.1	1.2, 2.1	
SERE, D				
Mean ± SD	−2.8 ± 5.1	−2.7 ± 5.3	−3.0 ± 4.7	0.8693
95% CI	−3.9, −1.7	−4.0, −1.4	−5.3, −0.6	
Anticoagulant/antiplatelet use				
Yes, *n* (%)	15 (18)	10 (16)	5 (24)	0.5097
No, *n* (%)	70 (82)	54 (84)	16 (76)	

*p* values were calculated between surgical groups using the unpaired *t* test for continuous data and Fisher’s exact probability test for categorical data. ** indicates a significance level of 1% (*p* < 0.01) for the *t*-test or Fisher’s exact probability test. CE, cataract extraction; POAG, primary open-angle glaucoma; EXG, exfoliation glaucoma; SERE, sphere equivalent refractive error; SD, standard deviation; CI, confidence interval; TMH, Tanito microhook trabeculotomy; IOP, intraocular pressure; D, diopters.

**Table 3:** IOP, medication, and RLC scores during follow-up.

Periods	Postop 1D		Postop 2D		Postop 3D		Postop 2W		Postop 3M	
Parameters	TMH-CE	iStent-CE	TMH-CE	iStent-CE	TMH-CE	iStent-CE	TMH-CE	iStent-CE	TMH-CE	iStent-CE
IOP, mmHg										
Mean ± SD	14.2 ± 7.3	10.7 ± 4.2	12.7 ± 5.7	9.4 ± 3.2	13.0 ± 7.8	9.7 ± 3.6	16.1 ± 7.2	14.1 ± 4.0	13.7 ± 4.7	13.6 ± 1.8
95% CI	12.3, 16.0	8.8, 13.0	11.3, 14.1	7.9, 10.8	11.1, 15.0	8.1, 11.4	14.3, 17.9	12.3, 16.0	12.5, 14.9	12.8, 14.4
*p* value		0.0449 *		0.0130 *		0.0656		0.2234		0.9362
Medications										
Mean ± SD	2.1 ± 0.9	1.5 ± 0.9	2.1 ± 0.9	1.5 ± 0.9	2.1 ± 0.9	1.4 ± 0.9	2.1 ± 0.9	1.4 ± 1.0	1.9 ± 0.9	1.4 ± 1.0
95% CI	1.8, 2.3	1.1, 1.9	1.9, 2.3	1.1, 1.9	1.8, 2.3	1.0, 1.8	1.9, 2.3	1.0, 1.9	1.7, 2.2	1.0, 1.9
*p* value		0.0112 *		0.0058 **		0.0060 **		0.0051 **		0.0432 *
R										
Mean ± SD	2.6 ± 0.6	1.0 ± 1.1	1.9 ± 0.9	0.4 ± 0.7	1.4 ± 0.9	0.0 ± 0.2	0.1 ± 0.4	0	0	0
95% CI	2.5, 2.8	0.5, 1.5	1.7, 2.2	0.0, 0.7	1.1, 1.6	−0.1, 0.1	−0.0, 0.2	0	0	0
*p* value		<0.0001 **		<0.0001 **		<0.0001 **		0.3182		
L										
Mean ± SD	0.3 ± 0.6	0.0 ± 0.2	0.1 ± 0.4	0.0 ± 0.2	0.0 ± 0.2	0	0	0	0	0
95% CI	0.2, 0.5	−0.1, 0.1	0.0, 0.2	−0.1, 0.1	−0.0, 0.1	0	0	0	0	0
*p* value		0.0296 *		0.3057		0.4184				
C										
Mean ± SD	0.3 ± 0.5	0.1 ± 0.3	0.3 ± 0.5	0.1 ± 0.3	0.2 ± 0.4	0.1 ± 0.3	0.0 ± 0.0	0	0	0
95% CI	0.2, 0.4	−0.0, 0.2	0.2, 0.4	−0.0, 0.2	0.1, 0.3	−0.0, 0.2	0.0, 0.1	0	0	0
*p* value		0.0642		0.0375 *		0.1705		0.3182		
RLC										
Mean ± SD	3.2 ± 1.1	1.1 ± 1.3	2.4 ± 1.3	0.5 ± 1.0	1.6 ± 1.3	0.1 ± 0.4	0.1 ± 0.6	0	0	0
95% CI	3.0, 3.5	0.6, 1.7	2.1, 2.7	0.1, 0.9	1.3, 1.9	0.0, 0.3	−0.0, 0.3	0	0	0
*p* value		<0.0001 **		<0.0001 **		<0.0001 **		0.2777		

*p* values were calculated between the surgical groups using the unpaired *t*-test. * *p* < 0.05, ** *p* < 0.01. 1D, 1 day; 2D, 2 days; 3D, 3 days; 2W, 2 weeks; 3M, 3 months; Postop, postoperative; TMH, Tanito microhook trabeculotomy; CE, cataract extraction; IOP, intraocular pressure; R, red blood cells score; L, layer formation score; C, blood clot score; RLC, total of R--L and C scores; SD, standard deviation; CI, confidence interval.

**Table 4:** Possible associations between RLC scores on postoperative day 1 and various continuous parameters.

Score	R		L		C		RLC	
Parameters	r (95% CI)	*p*-Value	r (95% CI)	*p*-Value	r (95% CI)	*p*-Value	r (95% CI)	*p*-Value
Age, years	−0.17 (−0.37, 0.05)	0.1246	−0.04 (−0.25, 0.18)	0.7260	−0.30 (−0.48, −0.09)	0.0058 **	−0.22 (−0.41, −0.01)	0.0423 *
Preoperative IOP, mmHg	0.25 (0.04, 0.44)	0.0195 *	0.08 (−0.13, 0.29)	0.4436	0.09 (−0.13, 0.30)	0.4237	0.23 (0.00, 0.43)	0.0307 *
Preoperative medications	0.26 (0.05, 0.45)	0.0165 *	0.28 (0.07, 0.46)	0.0099 **	0.04 (−0.17, 0.25)	0.7074	0.29 (0.09, 0.48)	0.0063 **
SERE, D	0.04 (−0.18, 0.26)	0.7166	0.16 (−0.06, 0.36)	0.1527	−0.29 (−0.48, −0.08)	0.0088 **	−0.00 (−0.22, 0.22)	0.9877
Postoperative 1D IOP, mmHg	0.21 (−0.00, 0.40)	0.0542	0.22 (0.01, 0.42)	0.0396 *	0.21 (−0.01, 0.40)	0.0576	0.29 (0.08, 0.47)	0.0073 **
Postoperative 1D ΔIOP, mmHg	0.13 (−1.34, 1.61)	0.8573	2.13 (−0.77, 5.04)	0.1480	2.22 (−1.23, 5.69)	0.2050	0.53 (−0.50, 1.57)	0.3093
Postoperative 1D medication	0.18 (−0.03, 0.38)	0.0970	0.19 (−0.02, 0.39)	0.0747	−0.07 (−0.28, 0.15)	0.5418	0.18 (−0.04, 0.38)	0.1056
Postoperative 1D Δmedication	−0.11 (−0.30, 0.08)	0.2564	−0.24 (−0.62, 0.15)	0.2224	−0.25 (−0.70, 0.21)	0.2844	−0.11 (−0.24, 0.03)	0.1191
Postoperative 3M IOP, mmHg	0.04 (−0.18, 0.25)	0.7265	−0.20 (−0.39, 0.02)	0.0732	0.24 (0.03, 0.43)	0.0250 *	0.03 (−0.18, 0.24)	0.7822
Postoperative 3M ΔIOP, mmHg	−1.11 (−2.29, 0.07)	0.0653	−2.41 (−4.75, −0.07)	0.0441 *	1.28 (−1.56, 4.12)	0.3723	−0.74 (−1.58, 0.09)	0.0814
Postoperative 3M medication	0.14 (−0.08, 0.34)	0.2064	0.20 (−0.01, 0.40)	0.0674	0.03 (−0.18, 0.25)	0.7586	0.18 (−0.04, 0.38)	0.1022
Postoperative 3M Δmedication	−0.14 (−0.37, 0.08)	0.2052	−0.21 (−0.66, 0.24)	0.3513	−0.03 (−0.56, 0.51)	0.9184	−0.10 (−0.26, 0.06)	0.2092

The correlation coefficient (r) was calculated using Pearson’s correlation coefficient test. * *p* < 0.05, ** *p* < 0.01. R, red blood cells score; L, layer formation score; C, blood clot score; RLC, total of R, L, and C scores; CI, confidence interval; IOP, intraocular pressure; SERE, sphere equivalent refractive error; Δ, change from baseline value; 3M, 3 months; 1D, 1 day; D, diopters.

**Table 5:** Possible associations between RLC scores on postoperative day 1 and various categorical parameters.

Parameters	Mean ± SD (95% CI)	Mean ± SD (95% CI)	*p*-Value
R			
Gender	Male, 2.2 ± 1.1 (1.8, 2.6)	Female, 2.2 ± 1.0 (1.9, 2.5)	0.9565
Glaucoma type	POAG, 2.1 ± 1.1 (1.8, 2.4)	EXG, 2.4 ± 0.9 (2.1, 2.8)	0.2088
Anticoagulant/antiplatelet use	Yes, 2.5 ± 0.5 (2.2, 2.8)	No, 2.1 ± 1.1 (1.9, 2.4)	0.1820
L			
Gender	Male, 0.4 ± 0.6 (0.2, 0.6)	Female, 0.2 ± 0.4 (0.0, 0.3)	0.0375 *
Glaucoma type	POAG, 0.2 ± 0.5 (0.1, 0.3)	EXG, 0.3 ± 0.6 (0.1, 0.6)	0.3024
Anticoagulant/antiplatelet use	Yes, 0.4 ± 0.5 (0.1, 0.7)	No, 0.2 ± 0.5 (0.1, 0.4)	0.2446
C			
Gender	Male, 0.2 ± 0.4 (0.1, 0.4)	Female, 0.3 ± 0.4 (0.1, 0.4)	0.7520
Glaucoma type	POAG, 0.2 ± 0.4 (0.1, 0.3)	EXG, 0.3 ± 0.5 (0.1, 0.5)	0.7565
Anticoagulant/antiplatelet use	Yes, 0.1 ± 0.4 (−0.1, 0.3)	No, 0.3 ± 0.4 (0.2, 0.4)	0.2658
RLC			
Gender	Male, 2.8 ± 1.6 (2.3, 3.4)	Female, 2.6 ± 1.3 (2.3, 3.0)	0.5509
Glaucoma type	POAG, 2.6 ± 1.5 (2.2, 3.0)	EXG, 3.0 ± 1.3 (2.5, 3.6)	0.1780
Anticoagulant/antiplatelet use	Yes, 3.1 ± 0.8 (2.6, 3.5)	No, 2.6 ± 1.6 (2.3, 3.0)	0.3080

*p* values were calculated using the unpaired *t*-test between groups. * *p* < 0.05. SD, standard deviation; CI, confidence interval; R, red blood cells score; L, layer formation score; C, blood clot score; RLC, total of R, L, and C scores; POAG, primary open-angle glaucoma; EXG, exfoliation glaucoma.

**Table 6:** Possible associations between postoperative day-1 RLC scores and various parameters analyzed by a multiple regression model.

Score	R		L		C		RLC	
Parameters	Estimates (95% CI)	*p*-Value	Estimates (95% CI)	*p*-Value	Estimates (95% CI)	*p*-Value	Estimates (95% CI)	*p*-Value
Gender (male)	0.05 (−0.14, 0.23)	0.5940	−0.08 (−0.20, 0.04)	0.1911	−0.03 (−0.13, 0.07)	0.5926	−0.06 (−0.35, 0.23)	0.6903
Glaucoma type (PG)	0.02 (−0.18, 0.22)	0.8640	−0.06 (−0.19, 0.07)	0.4176	0.02 (−0.09, 0.13)	0.7349	−0.03 (−0.34, 0.29)	0.8690
Anticoagulant/antiplatelet use (yes)	0.26 (0.02, 0.50)	0.0315 *	0.08 (−0.08, 0.23)	0.3385	−0.00 (−0.13, 0.12)	0.9498	0.33 (−0.04, 0.71)	0.0773
Surgical procedure (TMH-CE)	0.81 (0.57, 1.05)	<0.0001 **	0.06 (−0.09, 0.22)	0.4176	0.15 (0.02, 0.27)	0.0260 *	1.02 (0.64, 1.39)	<0.0001 **
Age, years	−0.00 (−0.03, 0.02)	0.8386	−0.00 (−0.02, 0.01)	0.8347	−0.01 (−0.02, 0.00)	0.1946	−0.01 (−0.05, 0.02)	0.5055
Preoperative IOP, mmHg	−0.01 (−0.05, 0.03)	0.7295	−0.01 (−0.04, 0.02)	0.4586	−0.01 (−0.03, 0.01)	0.4844	−0.03 (−0.09, 0.04)	0.4385
Preoperative medications	−0.11 (−0.34, 0.12)	0.3325	0.09 (−0.05, 0.25)	0.2165	−0.04 (−0.16, 0.08)	0.5363	−0.06 (−0.41, 0.30)	0.7568
SERE, D	0.00 (−0.03, 0.04)	0.9172	0.01 (−0.01, 0.04)	0.2933	−0.02 (−0.04, 0.00)	0.0271 *	−0.01 (−0.06, 0.05)	0.7954
Postoperative 1D IOP, mmHg	0.01 (−0.01, 0.04)	0.3590	0.01 (−0.00, 0.03)	0.1043	0.01 (−0.00, 0.02)	0.1870	0.04 (−0.01, 0.08)	0.0853
Postoperative 1D medications	0.04 (−0.21, 0.30)	0.7428	−0.01 (−0.18, 0.16)	0.9443	−0.06 (−0.20, 0.07)	0.3557	−0.03 (−0.43, 0.37)	0.8908
Postoperative 3M IOP, mmHg	0.01 (−0.03, 0.06)	0.5249	−0.03 (−0.06, 0.00)	0.0828	0.02 (−0.00, 0.04)	0.0927	0.01 (−0.06, 0.08)	0.8035
Postoperative 3M medications	−0.05 (−0.26, 0.17)	0.6434	0.02 (−0.12, 0.17)	0.7364	0.02 (−0.09, 0.14)	0.7072	−0.00 (−0.34, 0.33)	0.9797

*p* values were calculated using a multiple regression model. * p < 0.05, ** *p* < 0.01. IOP, intraocular pressure; PG, primary open-angle glaucoma group; TMH, Tanito microhook trabeculotomy; CE, cataract extraction; SERE, sphere equivalent refractive error; D, diopters; 1D, 1 day; 3M, 3 months; CI, confidence interval; R, red blood cells score; L, layer formation score; C, blood clot score; RLC, total of R, L, and C scores.

**Table 7:** Possible associations between postoperative day-1 RLC scores and various parameters analyzed by a multiple regression model in the TMH-CE group.

Score	R		L		C		RLC	
Parameters	Estimates (95% CI)	*p*-Value	Estimates (95% CI)	*p*-Value	Estimates (95% CI)	*p*-Value	Estimates (95% CI)	*p*-Value
Gender	−0.05 (−0.23, 0.13)	0.5594	−0.08 (−0.25, 0.08)	0.3008	−0.03 (−0.16, 0.10)	0.6143	−0.17 (−0.50, 0.16)	0.3107
Glaucoma type (PG)	0.00 (−0.17, 0.17)	0.9560	−0.07 (−0.22, 0.09)	0.3773	0.01 (−0.11, 0.14)	0.8169	−0.05 (−0.37, 0.27)	0.7519
Anticoagulant/antiplatelet use (yes)	−0.09 (−0.32, 0.15)	0.4742	0.04 (−0.18, 0.26)	0.7147	−0.04 (−0.21, 0.13)	0.6745	−0.08 (−0.52, 0.36)	0.7136
Age, years	−0.01 (−0.03, 0.01)	0.3776	0.00 (−0.02, 0.02)	0.8580	−0.01 (−0.03, 0.00)	0.1201	−0.02 (−0.06, 0.02)	0.2454
Preoperative IOP, mmHg	−0.01 (−0.04, 0.03)	0.7260	−0.01 (−0.04, 0.02)	0.5630	0.00 (−0.03, 0.02)	0.7477	−0.02 (−0.09, 0.05)	0.5514
Preoperative medications	−0.09 (−0.29, 0.11)	0.3556	0.11 (−0.07, 0.29)	0.2178	−0.04 (−0.18, 0.10)	0.6005	−0.02 (−0.39, 0.35)	0.9282
SERE, D	0.00 (−0.03, 0.04)	0.7913	0.01 (−0.02, 0.04)	0.3455	−0.03 (−0.05, −0.01)	0.0152 *	−0.01 (−0.07, 0.05)	0.7221
Postoperative 1D IOP, mmHg	0.02 (−0.01, 0.04)	0.1362	0.02 (−0.01, 0.04)	0.1442	0.01 (−0.01, 0.02)	0.3376	0.04 (−0.00, 0.08)	0.0612
Postoperative 1D medications	0.05 (−0.17, 0.27)	0.6459	0.01 (−0.19, 0.21)	0.9593	−0.05 (−0.20, 0.11)	0.5262	0.01 (−0.39, 0.41)	0.9666
Postoperative 3M IOP, mmHg	0.00 (−0.03, 0.04)	0.8106	−0.03 (−0.06, 0.01)	0.1213	0.02 (−0.01, 0.05)	0.1663	−0.00 (−0.07, 0.07)	0.9184
Postoperative 3M medications	−0.14 (−0.33, 0.05)	0.1582	0.03 (−0.15, 0.20)	0.7460	0.05 (−0.09, 0.19)	0.4828	−0.06 (−0.42, 0.30)	0.7384

*p* values were calculated using a multiple regression model. * *p* < 0.05. IOP, intraocular pressure; PG, primary open-angle glaucoma group; SERE, sphere equivalent refractive error; D, diopters; 1D, 1 day; 3M, 3 months; CI, confidence interval; R, red blood cells score; L, layer formation score; C, blood clot score; RLC, total of R, L, and C scores.

**Table 8:** Possible associations between postoperative 1D RLC scores and various parameters analyzed by a multiple regression model in iStent-CE group.

Score	R		L		C		RLC	
Parameters	Estimates (95% CI)	*p*-Value	Estimates (95% CI)	*p*-Value	Estimates (95% CI)	*p*-Value	Estimates (95% CI)	*p*-Value
Gender	−0.01 (−0.87, 0.85)	0.9780	0.00 (−0.24, 0.23)	0.9789	0.17 (0.03, 0.30)	0.0243	0.15 (−0.76, 1.06)	0.6966
Glaucoma type (PG)	0.12 (−1.65, 1.89)	0.8704	0.14 (−0.34, 0.63)	0.4976	0.11 (−0.17, 0.39)	0.3567	0.38 (−1.50, 2.26)	0.6391
Anticoagulant/antiplatelet use (yes)	1.04 (0.09, 1.98)	0.0362 *	0.02 (−0.24, 0.28)	0.8415	0.08 (−0.07, 0.23)	0.2552	1.13 (0.13, 2.14)	0.0326 *
Age, years	0.00 (−0.17, 0.17)	0.9676	0.02 (−0.02, 0.07)	0.2795	−0.01 (−0.04, 0.01)	0.2666	0.01 (−0.17, 0.19)	0.8751
Preoperative IOP, mmHg	−0.06 (−0.38, 0.26)	0.6569	0.03 (−0.06, 0.12)	0.4217	0.01 (−0.04, 0.06)	0.7797	−0.02 (−0.37, 0.32)	0.8675
Preoperative medication	−0.47 (−3.01, 2.07)	0.6660	0.09 (−0.60, 0.79)	0.7570	0.15 (−0.25, 0.55)	0.3890	−0.23 (−2.93, 2.48)	0.8438
SERE, D	0.03 (−0.29, 0.36)	0.8138	−0.05 (−0.13, 0.04)	0.2571	0.03 (−0.02, 0.08)	0.2439	0.01 (−0.33, 0.36)	0.9230
Postoperative 1D IOP, mmHg	−0.04 (−0.22, 0.13)	0.5535	−0.01 (−0.06, 0.03)	0.4897	0.04 (0.01, 0.06)	0.0201 *	−0.02 (−0.21, 0.16)	0.7640
Postoperative 1D medications	0.19 (−7.39, 7.77)	0.9526	−0.57 (−2.64, 1.50)	0.5266	−0.25 (−1.44, 0.95)	0.6314	−0.62 (−8.68, 7.44)	0.8560
Postoperative 3M IOP, mmHg	0.05 (−0.49, 0.58)	0.8402	−0.09 (−0.23, 0.06)	0.1940	−0.06 (−0.14, 0.03)	0.1440	−0.10 (−0.67, 0.47)	0.6843
Postoperative 3M medications	0.44 (−4.73, 5.61)	0.8425	0.46 (−0.96, 1.87)	0.4606	−0.07 (−0.89, 0.74)	0.8295	0.82 (−4.68, 6.31)	0.7281

*p* values were calculated using a multiple regression model. * *p* < 0.05. IOP, intraocular pressure; PG, primary open-angle glaucoma group; SERE, sphere equivalent refractive error; D, diopters; 1D, 1 day; 3M, 3 months; CI, confidence interval; R, red blood cells score; L, layer formation score; C, blood clot score; RLC, total of R, L, and C scores.

## Data Availability

Data are fully available upon reasonable request to the corresponding author.
